# Care of the Oral Cavity during Pregnancy

**Published:** 1904-04-15

**Authors:** C. H. Oakman


DENTAL REGISTER.

Vol. LVIII. APRIL 15, 1904. No. 4.

### CARE OF THE ORAL CAVITY DURING PREGNANCY.

BY C. H. OAKMAN, D.D.S.

Any disease affecting the general health, such as infective inflammatory or suppurative processes which influence bodily nutrition, is likely to affect the dental organs, as they are more prone to decay than other tissues. Especially is this so during pregnancy, when the lime salts are taken from the mother for the upbuilding of the fœtus. To obviate this, some authors claim that the administration of the calcic phosphates prevents this tendency. There is nothing very definite about their use clinically. Some claim that lime salts taken into the system in such a manner also leaves the system as such, not being assimilated, but to be sure that the lime salts are taken up by the system they should be taken in the form of food, such as whole wheat bread, cereals, etc.

It is supposed by many that the teeth should receive no operative treatment during pregnancy. I believe that many cases have received judicious treatment during this period, and have been benefited without hazard to the patient. It would be unwise to allow a woman to suffer with neuralgia, toothache, abscess, carious bone or diseased antrum, which are liable to intense pain or suppurative conditions, which may affect the general health. Indigestion or gastric intestinal conditions are provoked by pus absorption, and the suffering and inconvenience which a woman would ordinarily endure, if these conditions were to continue, would to my mind be as great a tax on the nervous system as the stages of labor. Loss of sleep, nausea, and irritability would be accentuated by constant or frequent attacks of pain, consequently weakening the system and possibly inducing miscarriage.

As a rule, only temporary operations should be performed on the teeth, especially if the patient is of the neurasthenic type. Some women claim that they never feel better than when pregnant, in such cases permanent work might be done with impunity, providing the teeth are not excessively sensitive, and can be filled without much inconvenience. The greatest care and tact are often necessary to accomplish the simplest treatment for some women; consequently any attempt to prolong the sittings, as is often necessary in filling teeth, would result in failure of the operation, and possibly with disastrous results. All diseased teeth, which cannot be saved and which would irritate or abscess, should be removed under an anesthetic to avoid any great shock.

It is a well-known fact that the saliva of a pregnant woman is acid, as a result of which we often get chemical erosions of the necks of the teeth, which become very sensitive to the touch, their structure breaking down and producing very painful cavities.

This condition is often accompanied by pyorrhœa alveolaris with pus oozing around the necks of the teeth. The gums are œdematous, congested and of a purple color. The treatment is to remove all calcareous deposits and to irrigate the teeth and all pus pockets with warm boric acid solution or a hot solution of chloretone applied by a syringe. Hydrogen dioxide is contra-indicated on account of its acid reaction. Milk of magnesia, lime water, glycothymoline or soda water are valuable agents on account of their alkalinity. The majority of mouth-washes are not alkaline, in fact their so-called cleaning properties depend on the acids they contain. All such washes should be discarded, and especially those containing formaldehyde.

It should be borne in mind that the teeth should be cleaned mechanically and not chemically, and for this reason a good tooth-powder is preferable to the so-called mouthwashes. If only washes are used, debris will accumulate on the buccal surfaces, a recession of the gum will occur—or an inflamatory tumefaction of the gums will take place—due to the lack of friction which the brush would furnish.

The teeth should be brushed with powder after each meal and at night. It is safe to say that not one pregnant woman in five hundred brushes her teeth correctly or efficiently. The brush handle should be grasped in the palm of the hand, not exactly a "Prague" grasp, but a firm hold, with the powder on the dampened brush; it should be inserted into the posterior buccal region, and over the surfaces of the upper molars, and the brush brought downward by rotating the wrist, striking the gum before the teeth. The procedure is just the reverse on the lower jaw. The grinding surface should also be carefully brushed from before backward silk floss should be passed between the teeth daily.

It is advisable, during gestation, that an alkaline mouth wash should be used several times a day, as it corrects acidity and has an inhibitory action on the multiplication of the caries-producing influences. Milk of magnesia is most excellent, as in addition to its alkaline reaction, it produces a protective film that will last several hours; this is especially indicated on retiring when the functions of the secretory glands are at rest. The consumption of sweets should be limited during pregnancy, as its tendency is to increase the acid fermentation in the mouth.

Stomatitis is frequently met with as a complication of pregnancy, its treatment being constitutional, with the local application of mild antiseptics, chlorate of potash solution or cauterant, silver nitrate if necessary.

In prescribing tonics containing iron or hydrochloric acid, care should be taken to warn the patient to use an alkaline mouth-wash after taking, as many teeth have been injured or lost through the careless administration of such drugs.

If an abscess from a diseased tooth should appear during pregnancy, have the patient consult the dentist, as simply lancing of the gums, as is usually done by the physician, is often insufficient for permanent relief. Infection frequently extends from a putrescent pulp to adjacent tissues. In this condition poultices should never be applied to the face, as they favor suppuration of the buccal structures and necrosis of the bones, resulting in a permanent disfigurement of the face.

Extensive artificial work, which would irritate or prostrate the patient, should never be attempted during pregnancy; all therapeutic measures should be palliative rather than radical, the object being to avoid shock to a system which is already hyper-sensitive. If the occasion demands an operation which entails more or less pain and would result in shock, we would employ local or profound general anesthetics.

Extraction of teeth must not be performed indiscriminately, and before attempting any operative measures; we should enquire into the physical and nervous condition as well as the temperament of the patient. It is needless to say that all manipulations should be performed with the utmost gentleness; steel instruments should be warmed, and all unnecessary exhibition of them avoided on account of psychic influences.

